# Modulation of Statin-Activated Shedding of Alzheimer APP Ectodomain by ROCK

**DOI:** 10.1371/journal.pmed.0020018

**Published:** 2005-01-11

**Authors:** Steve Pedrini, Troy L Carter, George Prendergast, Suzana Petanceska, Michelle E Ehrlich, Sam Gandy

**Affiliations:** 1Farber Institute for Neurosciences, Thomas Jefferson UniversityPhiladelphia, PennsylvaniaUnited States of America; **2**Lankenau Institute for Medical Research, WynnewoodPennsylvaniaUnited States of America; **3**Nathan S. Kline Institute for Psychiatric Research, Department of PsychiatryNew York University School of Medicine, Orangeburg, New YorkUnited States of America; Karolinska InstituteSweden

## Abstract

**Background:**

Statins are widely used cholesterol-lowering drugs that act by inhibiting HMGCoA reductase, the rate-limiting enzyme in cholesterol biosynthesis. Recent evidence suggests that statin use may be associated with a decreased risk for Alzheimer disease, although the mechanisms underlying this apparent risk reduction are poorly understood. One popular hypothesis for statin action is related to the drugs' ability to activate α-secretase-type shedding of the α-secretase-cleaved soluble Alzheimer amyloid precursor protein ectodomain (sAPP_α_). Statins also inhibit the isoprenoid pathway, thereby modulating the activities of the Rho family of small GTPases—Rho A, B, and C—as well as the activities of Rac and cdc42. Rho proteins, in turn, exert many of their effects via Rho-associated protein kinases (ROCKs). Several cell-surface molecules are substrates for activated α-secretase-type ectodomain shedding, and regulation of shedding typically occurs via activation of protein kinase C or extracellular-signal-regulated protein kinases, or via inactivation of protein phosphatase 1 or 2A. However, the possibility that these enzymes play a role in statin-stimulated shedding has been excluded, leading us to investigate whether the Rho/ROCK1 protein phosphorylation pathway might be involved.

**Methods and Findings:**

We found that both atorvastatin and simvastatin stimulated sAPP_α_ shedding from a neuroblastoma cell line via a subcellular mechanism apparently located upstream of endocytosis. A farnesyl transferase inhibitor also increased sAPP_α_ shedding, as did a dominant negative form of ROCK1. Most conclusively, a constitutively active ROCK1 molecule inhibited statin-stimulated sAPP_α_ shedding.

**Conclusion:**

Together, these data suggest that statins exert their effects on shedding of sAPP_α_ from cultured cells, at least in part, by modulation of the isoprenoid pathway and ROCK1.

## Abstract 2

The cholesterol-lowering statins seem to have a protective effect against Alzheimer disease. In cultured cells, statins affect cleavage of the amyloid precursor protein via the ROCK kinase. This could explain some of their beneficial effects

## Introduction

Alzheimer disease is the leading cause of dementia among the elderly and is characterized by accumulation of extracellular and vascular amyloid in the brain [1]. Amyloid deposits are composed of the amyloid-β peptide (Aβ), a 4-kDa peptide released during “amyloidogenic” proteolytic processing of the Alzheimer Aβ precursor protein (APP) [2]. APP can also be cleaved by the nonamyloidogenic α-secretases, a disintegrin and metalloproteinase 10 (ADAM-10) and ADAM-17 [3], in a reaction that is believed to occur primarily on the plasma membrane [4] and is known as “ectodomain shedding.” α-Secretase-type ectodomain shedding divides the Aβ domain of APP, thereby generating α-secretase-cleaved soluble APP ectodomain (sAPP_α_) [4]. This reaction can be stimulated by activation of protein kinase C (PKC) or extracellular-signal-regulated protein kinases (ERKs) [5,6,7] or by inactivation of protein phosphatase 1 or 2A [5].

Reports from retrospective analyses suggest that the statin class of cholesterol-lowering HMGCoA reductase inhibitors may lower the risk for Alzheimer disease by as much as 70% [8,9,10,11]. Studies in wild-type guinea pigs and in plaque-forming transgenic mice have demonstrated that chronic statin treatment can attenuate cerebral amyloidosis [12,13], suggesting that statins may exert their risk-reducing effects, at least in part, by modulating APP metabolism. In cell culture, lovastatin and simvastatin decrease the release of Aβ by rat hippocampal neurons [12,14] while activating α-secretase-type ectodomain shedding [15,16]. However, the molecular mechanisms by which statins modulate ectodomain shedding remain to be elucidated [17,18].

Statin effects on APP metabolism are, to some extent, attributable to cholesterol lowering, but statin actions on APP may also involve cholesterol-independent actions [19]. Reduction in synthesis of mevalonate leads to decreased generation of a number of isoprenoid lipid derivatives. Isoprenoids, such as farnesyl pyrophosphate and geranylgeranyl pyrophosphate, are 15- or 20-carbon lipid moieties. Through the action of farnesyl transferases and type I geranylgeranyl transferases, isoprenoids are attached to the amino acid sequence Cys-Ala-Ala-Xaa (“CAAX”) at the C-terminus of the Rho family of GTPases [20]. These posttranslational lipid modifications are essential for attachment of the GTPases to the cytosolic face of intracellular vesicles and/or to the cytosolic leaflet of the plasma membrane, thereby specifying subcellular targets for GTPase action(s).

Some members of the Rho GTPase family exert their actions through modulation of protein kinase activities. One of the best characterized is Rho-associated protein kinase 1 (ROCK1, also called ROKβ). ROCK1 is a serine/threonine kinase with an apparent mass of 160 kDa that can be activated by either RhoA or RhoB [21,22,23]. Structurally, the ROCK1 N-terminus contains the protein kinase domain, while the C-terminus has both a Rho-binding domain and a pleckstrin homology domain, either of which can modulate protein–protein interactions. In the inactive state, the Rho-binding domain and the pleckstrin homology domain form an autoinhibitory loop by binding and blocking the kinase domain at the N-terminus of the molecule. Activation of ROCK1 occurs when a Rho protein binds to the Rho-binding domain, causing a conformational change that opens the kinase domain for the phosphorylation of downstream effectors [23]. Once activated, ROCK1 phosphorylates several substrates, including myosin light chain phosphatase, LIM kinases (Lin11, Isl1, and Mec3), and ezrin-radixin-moesin proteins [24,25,26,27]. ROCK1 has recently been implicated in modifying the site of substrate cleavage by APP γ-secretase [28], perhaps acting via ROCK1-dependent phosphorylation of a component of the γ-secretase enzyme complex.

In the current study, we demonstrate that activation of sAPP_α_ shedding from cultured cells by atorvastatin or simvastatin involves isoprenoid-mediated protein phosphorylation. Treatment of cells with a farnesyl transferase inhibitor or expression of a dominant negative (DN) ROCK1 molecule led to enhanced sAPP_α_ shedding, supporting the notion that shedding is modulated by the isoprenoid pathway. Transfection with the cDNA for a constitutively active (CA) ROCK1 molecule led to inhibition of statin-activated sAPP_α_ shedding. These results raise the possibility that the apparent beneficial effect of statins in the prevention of Alzheimer disease could be, at least in part, mediated by isoprenoid modulation of APP metabolism.

## Methods

### Reagents

The APP C-terminal specific polyclonal antibody 369 [29] was used to detect full-length APP and its C-terminal fragments. Monoclonal antibody 6E10 against residues 1–16 of human Aβ (Signet, Dedham, Massachusetts, United States) was used to detect human holoAPP or sAPP_α_. Anti-ROCK1 antibody was purchased from Chemicon (Temecula, California, United States). Streptavidin-antibody HRP-conjugated C-Myc antibody 9E10, mevalonic acid, arachidonic acid, and phenylarsine oxide were purchased from Sigma (St. Louis, Missouri, United States). Atorvastatin was obtained from Pfizer (Groton, Connecticut, United States), and simvastatin was obtained from LKT Labs (St. Paul, Minnesota, United States). N2 supplement was obtained from Gibco (Carlsbad, California, United States). Sulfo-NHS-LC-Biotin was purchased from Pierce (Rockford, Illinois, United States). CA and DN Myc-tagged ROCK1 vectors were generated as previously described [30,31] and were generous gifts from Liqun Luo (Stanford University). Fugene 6 was purchased from Roche (Basel, Switzerland). Farnesyl transferase inhibitor 1 (FTI-1) was obtained from Biomol (Plymouth Meeting, Pennsylvania, United States). Tumor necrosis factor α (TNFα) protease inhibitor 2 and Y-27632 were purchased from Calbiochem (San Diego, California, United States). Protein concentration assay kit was purchased from Biorad (Hercules, California, United States). LIVE/DEAD Viability/Cytotoxicity Assay Kits and Amplex Red Cholesterol Assay Kits were purchased from Molecular Probes (Eugene, Oregon, United States).

### Culture Methods and Sample Preparation

N2a mouse neuroblastoma cells stably transfected with the Swedish mutant form of APP (SweAPP N2a cells; APP695, 595–596 KM/NL) (gift from G. Thinakaran and S. Sisodia, University of Chicago, Chicago, Illinois, United States) were maintained in DMEM, 10% FBS, and 200 μg/ml G418 in the presence of penicillin and streptomycin [32]. For the 24 h prior to pharmacological treatments, the culture media were changed to N2-supplemented lipid-free medium. In some studies, cells were transfected in N2-supplemented FBS-free medium 48 h before pharmacological treatments. Transfections were carried out using the Fugene reagent, according to the manufacturer's instructions. All treatments were performed in the presence of 1 μM mevalonic acid, unless otherwise specified.

Cells were lysed in 1% Triton-X/PBS buffer containing 1 X complete proteinase inhibitor cocktail (Roche), sonicated twice for 30 s, and centrifuged at 5,000*g* for 5 min. Protein concentration in the supernatant was determined using the Biorad Protein Assay kit, following the manufacturer's instructions. For the detection of holoAPP and sAPP_α_, samples were separated in 7.5% polyacrylamide gels, transferred to nitrocellulose, and the proteins detected with either 369 (1:3,000 for holoAPP and C-terminal fragments) or 6E10 (1:1,000 for holoAPP or sAPP_α_), followed by incubation of the transfers with appropriate secondary anti-rabbit or anti-mouse antibodies. For the detection of transfected ROCK1 proteins, samples were immunoprecipitated with 2 μg of anti-Myc antibody, separated in 5% polyacrylamide gels, transferred, and the proteins detected with anti-ROCK1 antibody (1:1,000 dilution).

### Cell-Surface Biotinylation

Cells were plated in a 100-mm dish at a concentration of 5 × 10^6^ cells/dish. After treatment, media were harvested, and sAPP_α_ levels were evaluated by immunoblotting as described above. Cells were washed twice in PBS and then incubated with Sulfo-NHS-LC-Biotin for 30 min at 4 ^o^C. Biotinylation reactions were terminated by one wash in Tris followed by two washes in PBS. Cells were lysed in 1% Triton-X/PBS buffer containing protease inhibitor cocktail as indicated above. Lysates were immunoprecipitated with 3 μl of whole 369 antibody serum and 30 μl of protein A beads. After washing twice with 1% Triton/PBS, and then twice with PBS, samples were boiled in sample buffer for 3 min, separated in a 7.5% polyacrylamide gel, and transferred to nitrocellulose. The biotinylated proteins were detected using streptavidin HRP polymer (1:10,000 dilution).

### Viability/Cytotoxicity Assays

Cells were plated in an eight-well slide at a concentration of 1 × 10^4^ cells/well. After treatments as indicated, LIVE/DEAD assays were performed following the manufacturer's instructions (Molecular Probes).

### Cholesterol Assays

Cholesterol levels in cell lysates were measured using Amplex Red following the manufacturer's instructions (Molecular Probes). We have previously demonstrated that standard doses of either simvastatin or atorvastatin reduce cholesterol levels in N2a cells by 65%–67% [16].

### Quantification and Statistical Analysis

Quantification of protein bands was performed using the UVP Bioimaging System, and statistical analysis was performed on paired observations using the Student's *t* test.

## Results

### Atorvastatin Activates sAPP_α_ Shedding at a Subcellular Site Upstream of Endocytosis from the Plasma Membrane

We confirmed our previous observation [16] that atorvastatin produces an increase in sAPP_α_ shedding that is dose-dependent, reaching a maximum effect at 5 μM. The increase in sAPP_α_ shedding is accompanied by a corresponding increase in levels of the nonamyloidogenic APP α-C-terminal fragment (C83; data not shown).

**Figure 1:**
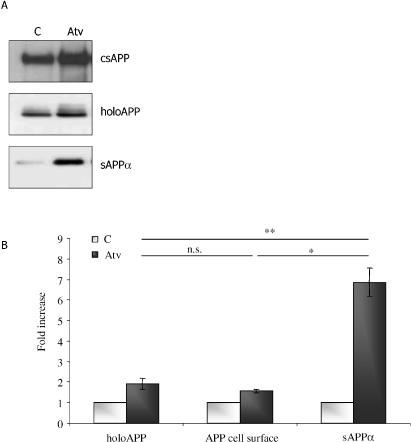
Atorvastatin Activates sAPP_α_ Shedding Out of Proportion to Its Effect on holoAPP or csAPP (A) SweAPP N2a cells were treated with atorvastatin (Atv) for 24 h and then surface biotinylation was performed as described in Methods. Evaluation of csAPP was performed by immunoprecipitation–immunoblot after surface biotinylation, while holoAPP and sAPP_α_ were evaluated by immunoblot as described in Methods. C, control. (B) Graphic representation of data. Y-axis shows effect of treatment (in arbitrary units) divided by effect of untreated control (in arbitrary units); *n* = 3 independent experiments; *, *p* < 0.05; **, *p* < 0.01; Student's *t* test).

In order to refine our localization of the subcellular target of statin action, we evaluated the steady-state levels of cell-surface APP (csAPP) in the absence or presence of statins. After drug treatments, cells were subjected to the surface biotinylation protocol. Cells and media were harvested, and levels of sAPP_α_, holoAPP, and csAPP were measured. Treatment with atorvastatin increased csAPP by approximately 1.6-fold, similar to the effect of the drug on holoAPP (Figure 1), while sAPP_α_ shedding was increased by approximately 7-fold (*p* < 0.05). Since csAPP levels were only slightly raised in the same statin-treated cells in which sAPP_α_ shedding was dramatically increased, we interpret this disparity to indicate that the effector of statin-stimulated shedding is probably intrinsic to the plasma membrane. In other studies, the plasma membrane has been proposed to be, or to contain, the statin target. For example, statins have been proposed to cause co-localization of α-secretase and APP within lipid rafts [15,33]; statins might also induce modification of the structure and activity of a protein in the plasma membrane α-secretase complex, perhaps in an action similar to how statins bind and “lock” cell-surface integrins [34].

A pulse-chase protocol was also used to study post-transcriptional regulation of APP metabolsm by statins (data not shown). This protocol avoids any confound that might arise because of altered APP transcription. Pulse-chase studies were performed using a 10-min pulse with [^35^S]methionine followed by various chase times from 0 to 120 min. Typical maturation and half-life of mature cellular holoAPP were observed, as was subsequent release of sAPP_α_ [5,29]. In the presence of either atorvastatin or simvastatin, the time course of maturation and release perfectly paralleled that observed in the absence of either drug, except that the fractional content of cellular mature holoAPP was approximately 2-fold greater in the presence of drug (i.e., at 15 or 75 min chase, mature APP in the presence of statin was approximately 310% of the level of immature APP at *t* = 0 versus a control [vehicle treatment] of 150% of the level of immature APP at *t* = 0; also, at *t* = 30 min, the relative percent values for drug versus vehicle were 380% and 200%, respectively). Fold increases in released sAPP_α_ in the same experiments were approximately 3- to 4-fold (2.0 arbitrary units versus 5.5–8.0 arbitrary units at 120 min for atorvastatin and simvastatin, respectively). Secretory maturation toxicity is one possible mechanism for elevated levels of intracellular mature holoAPP and causes retarded conversion of mature holoAPP to sAPP_α_. This pattern was not observed following statin treatment, excluding maturation toxicity as a mechanism underlying the altered levels of mature cellular holoAPP.

Instead, the pattern that we observed raises the possibility that statins, presumably via isoprenoids (given the reversibility with mevalonate), as discussed in the next section, may alter sorting of cellular holoAPP, diverting holoproteins away from terminal degradation in the endosomal/lysosomal pathway and into the constitutive secretory pathway that generates sAPP_α_. However, the fold effect on reduced intracellular turnover in the endosomal/lysosomal pathway (or sorting out of the endosomal/lysosomal pathway and into the constitutive secretory/shedding pathway) is apparently insufficient to explain the fold effect on sAPP_α_ generation (2-fold for the former, vs 3- to 4-fold for the latter), indicating a contribution from a downstream site in the processing pathway. When these results are taken together with independent work on regulated shedding of transforming growth factor α (TGFα) [35,36], a parsimonious explanation is that an important target for activation of ectodomain shedding is probably located at the plasma membrane or downstream of APP residence at the plasma membrane.

The identification of the regulatory components of the ectodomain shedding machinery have been long-sought in other studies employing phorbol esters to stimulate shedding of sAPP_α_ or TGFα [4,35,36,37]. Munc-13 has recently been implicated as a phorbol target in regulated shedding [38]. In our opinion, this molecule is rather unlikely to play a major role in shedding regulation, given the specificity of Munc-13 effects for phorbols and the generalization of the regulated shedding phenomenon to include activation by protein phosphatase inhibitors and neurotransmitters. Neither of these would be predicted to act via the phorbol-binding C1 domain of Munc-13.

**Figure 2:**
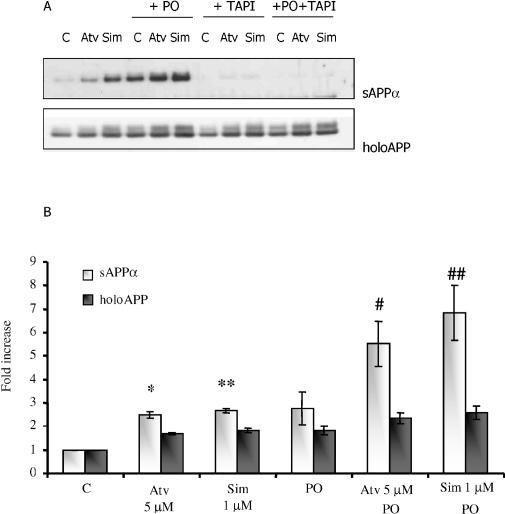
Simultaneous Treatment of SweAPP N2a Cells with Statins and an Inhibitor of Endocytosis (PO) Yields More sAPP_α_ Shedding Than Does Treatment with Either Statins or PO Alone (A) SweAPP N2a cells were treated for 24 h with atorvastatin (Atv) or simvastatin (Sim) as indicated. Media were then replaced and cells were treated for an additional 20 min with atorvastatin, simvastatin, TNFα protease inhibitor, PO, or combinations, as indicated. Evaluation of sAPP_α_ and holoAPP was performed by Western blot as described in Methods. C, control. (B) Graphic representation of data. Y-axis shows effect of treatment (in arbitrary units) divided by effect of untreated control (in arbitrary units); *n* = 3 independent experiments; *, *p* < 0.05 versus control; **, *p* < 0.01 versus C; #, *p* < 0.05 versus atorvastatin alone; ##, *p* < 0.05 versus simvastatin alone; Student's *t* test).

Sisodia and colleagues [39] demonstrated that arrest of APP endocytosis from the plasma membrane by deletion of its *NPXY* clathrin-coated vesicle targeting sequence [40,41] can dramatically stimulate sAPP_α_ shedding, presumably by extending the half-life of co-localized α-secretase and APP on the plasma membrane. In order to exclude the possible contribution of altered endocytosis to statin-stimulated shedding, we evaluated the effect of phenylarsine oxide (PO), an inhibitor of endocytosis, on statin-stimulated shedding (Figure 2). Treatment with either atorvastatin, simvastatin, or PO alone increased sAPP_α_ shedding, as expected. Co-treatment of cells with PO plus either atorvastatin or simvastatin caused stimulation of sAPP_α_ shedding to levels greater than the maximal levels of shedding achievable with inhibition of endocytosis using PO alone or with maximal doses of either statin alone (*p* < 0.05). The additivity of statin- and PO-stimulated shedding is consistent with the hypothesis that statins act at or near the plasma membrane, prior to internalization of csAPP. Under all circumstances, stimulated sAPP_α_ shedding was completely blocked using TNFα protease inhibitor 2, a standard α-secretase/metalloproteinase inhibitor (Figure 2). We interpret this as an indication that the statin-induced α-cleavage of APP is probably mediated by one of the molecules usually associated with the phenomenon, i.e., ADAM-10 or ADAM-17 [16].

### Compounds that Modulate Isoprenoid Levels Activate sAPP_α_ Shedding

**Figure 3:**
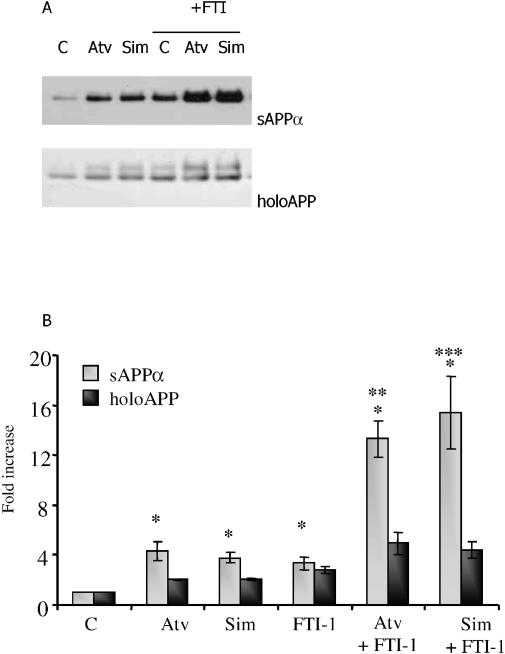
Simultaneous Treatment of SweAPP N2a Cells with a Statin and FTI-1 Causes Greater sAPP_α_ Shedding Than Either Drug Alone (A) SweAPP N2a cells were treated for 24 h with atorvastatin (Atv, 5 μM), simvastatin (Sim, 1 μM), FTI-1 (5 μM), or a combination of FTI-1 plus a statin. Levels of sAPP_α_ (top panel) or holoAPP (bottom panel) were evaluated as described in Methods. C, control. (B) Graphic representation of data. Y-axis shows effect of treatment (in arbitrary units) divided by effect of untreated control (in arbitrary units); *n* = 3 independent experiments; *, *p* < 0.05 versus control; **, *p* < 0.05 versus atorvastatin alone; ***, *p* < 0.05 versus simvastatin alone; Student's *t* test).

As discussed above, there is an established relationship between statins and isoprenoid-modulated protein phosphorylation. We therefore tested the effects of FTI-1 on statin-stimulated sAPP_α_ shedding. FTI-1 increased the shedding of sAPP_α_, but the combination of a statin plus FTI-1 increased sAPP_α_ shedding to levels greater than those achievable by using either compound alone (Figure 3; *p* < 0.05). In the same experiment, levels of holoAPP were modestly increased but, again, to an extent insufficient to account for the increase in shed sAPP_α_ (Figure 3).

**Figure 4:**
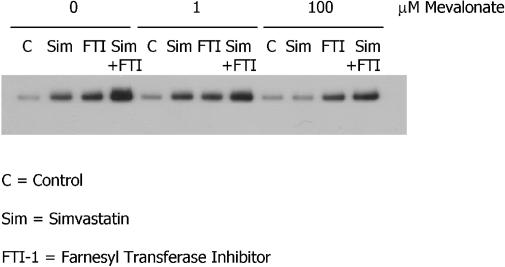
Mevalonic Acid Reverses Statin-Induced, but Not FTI-1-Induced, sAPP_α_ Shedding SweAPP N2a cells were treated for 24 h with simvastatin (Sim, 1 μM), FTI-1 (5 μM), mevalonic acid (0–100 μM), or combinations as indicated. Levels of sAPP_α_ were evaluated by western blot as described in Methods. This figure is representative of the results of two independent experiments. C, control.

To test whether statin-activated shedding might be attributable to a metabolite downstream of HMGCoA reductase, cells were treated with 1 μM simvastatin, 5 μM FTI-1, and a series of concentrations of mevalonic acid. Since FTI-1 acts downstream of HMGCoA reductase, the stimulatory effect of FTI-1 on sAPP_α_ shedding would not be predicted to be modified by mevalonate supplementation. Low doses (<1 μM) of mevalonic acid did not affect statin-induced sAPP_α_ shedding, but complete inhibition of statin-activated sAPP_α_ shedding was achieved with higher doses of mevalonic acid (100 μM). As predicted, the shedding observed following treatment with FTI-1 was not inhibited by any of the concentrations of mevalonic acid tested (Figure 4). These data are consistent with a role for isoprenoids in statin control of APP metabolism in cultured cells.

### Expression of ROCK-Related Molecules Modulates sAPP_α_ Shedding in a Bidirectional Manner

**Figure 5:**
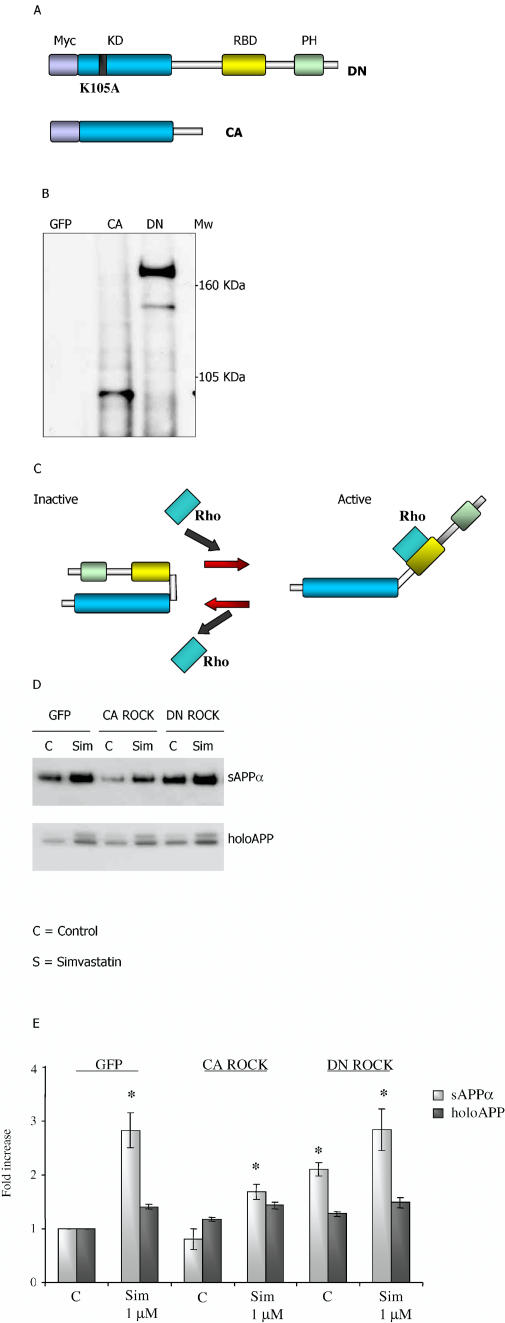
Structure and Expression of ROCK cDNAs, and Their Effect on Basal and Statin-Stimulated sAPP_α_ Shedding (A) Graphic representation of the ROCK1 constructs. Myc, Myc tag; KD, kinase domain; PH domain, pleckstrin homology domain; RBD, Rho-binding domain. (B) SweAPP N2a cells were transfected with GFP, CA ROCK1, or DN ROCK1 for 48 h. Cells were lysed and levels of expressed ROCK1 protein evaluated by immunoprecipitation–immunoblot as described in Methods. (C) Model for ROCK activity modulation by Rho. (D) SweAPP N2a cells were transfected for 48 h with control (GFP), CA ROCK1, or DN ROCK1 cDNAs. At the end of this incubation, cells were treated for an additional 24 h with simvastatin (Sim, 1 μM). sAPP_α_ and holoAPP were evaluated by immunoblot as described in Methods. (E) Graphic representation of data. Y-axis shows effect of treatment (in arbitrary units) divided by effect of untreated control (in arbitrary units); *n* = 3 independent experiments; *, *p* < 0.05 versus GFP; Student's *t* test). C, control.

**Figure 6:**
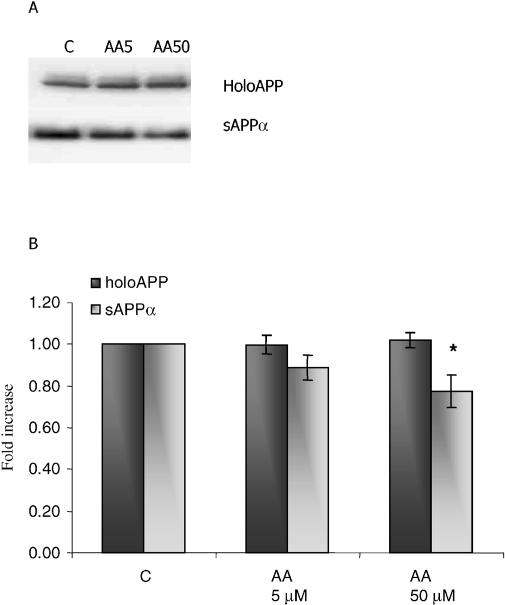
Arachidonic Acid Inhibits Basal sAPP_α_ Shedding but Has No Effect on holoAPP Levels (A) SweAPP cells were treated for 24 h with arachidonic acid (5 or 50 μM, represented by AA5 and AA50, respectively). Levels of sAPP_α_ were evaluated by immunoblot as described in Methods. C, control. (B) Graphic representation of data. Y-axis shows effect of treatment (in arbitrary units) divided by effect of untreated control (in arbitrary units); *n* = 6 independent experiments; *, *p* < 0.05 versus control; Student's *t* test.

Since many isoprenoid-mediated Rho effects converge on ROCKs, we next transfected N2a cells with cDNAs encoding either green fluorescent protein (GFP) (control), CA ROCK1, or DN ROCK1 (Figure 5). Simvastatin caused a typical activation of sAPP_α_ shedding from GFP-transfected cells. When CA ROCK1 was introduced, however, shedding of sAPP_α_ from both untreated and simvastatin-treated cells was diminished (Figure 5; *p* < 0.05 versus GFP control). Conversely, DN ROCK1 alone activated shedding of sAPP_α_. Cellular levels of holoAPP were not affected by transfection (Figure 5; *p* < 0.05 versus GFP control). In studies aimed at independent confirmation of the involvement of ROCK activation in sAPP_α_ shedding, we treated SweAPP N2a cells with arachidonic acid, an activator of ROCK. As shown in Figure 6, arachidonic acid reduced the shedding of sAPP_α_ without altering levels of holoAPP. Based on this series of results, we concluded that both basal and activated sAPP_α_ shedding from cultured cells are controlled by ROCK activity.

In some experiments, cells were treated with Y-27632 (10 nM to 50 μM), a compound that can inhibit ROCKs. Y-27632 showed no effect on basal sAPP_α_ release and blocked statin-activated sAPP_α_ shedding (data not shown). This result was unexpected in light of the effects of DN ROCK1. Given the internally consistent actions of DN ROCK1 and CA ROCK1, as well as the results employing either FTI-1 or arachidonate, we concluded that the Y-27632 result might be due to inhibition by Y-27632 of protein kinases other than ROCK1 [23]. The possibility was also considered that cytotoxicity of Y-27632 for the central vacuolar pathway might explain the disparity between the effects of DN ROCK1 and those of Y-27632, but neither impairment of intracellular APP maturation nor increased apoptosis as measured by LIVE/DEAD assay were apparent following Y-27632 treatment (data not shown). Ultimately, we were unable to document any explanation for the disparate results of Y-27632 and DN ROCK1.

## Discussion

**Figure 7:**
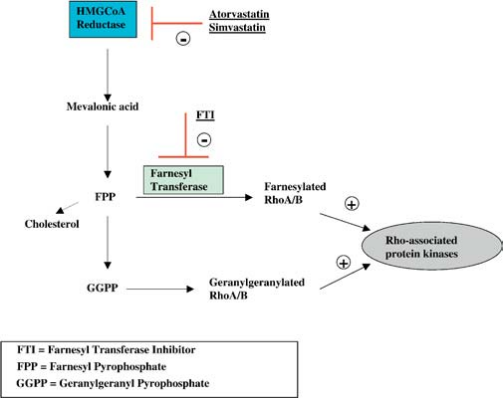
Isoprenoid Pathway and Sites of Action of Compounds Used in This Study FPP, farnesyl pyrophosphate; GGPP, geranylgeranyl pyrophosphate.

The isoprenoid pathway involves lipid modification of various members of the Rho family of small GTPases by the addition of either farnesyl or geranylgeranyl moieties [20,42]. Isoprenylation serves to target the GTPases to the proper organelle membrane, where their actions often relate to cytoskeletal dynamics and/or vesicle trafficking [20,42]. ROCKs are important downstream targets of Rho (Figure 7), catalyzing the phosphorylation of effector phosphoprotein substrates [23]. The foregoing data indicate that statin-induced activation of APP shedding in cultured cells involves the Rho/ROCK pathway. More specifically, the data indicate that ROCK1 activation blocks the effects of statins on APP ectodomain shedding, while ROCK1 blockade alone can mimic the effect of statins on APP shedding. By extension, these data predict that application of statins to neurons might directly or indirectly inhibit ROCK1 activity. Evaluation of this possibility will be the subject of future investigation.

The first evidence that APP might be a substrate for ectodomain shedding was provided by Weidemann et al. [43] who identified sAPP_α_ in the cerebrospinal fluid and blood. This aspect of APP metabolism bears resemblance to the proteolytic signal transduction pathways involved in processing pro-TGFα [35,36] and Notch [44]. In the case of Notch, the process is set in motion by the binding of a ligand to the Notch ectodomain, triggering its release (shedding). For APP, intracellular signal transduction appears to be more important [2,29,45]. In early studies, the existence of the shed ectodomain of APP was used to deduce the existence of the proteolytic activity designated α-secretase, which has the unusual specificity of cleaving its substrates at a proscribed distance from the extracellular leaflet of the plasma membrane [4,39]. Ultimately, the integral cell-surface metalloproteinases ADAM-10 and ADAM-17/TACE were found to underlie α-secretase-type ectodomain shedding [3,46].

Why is APP a substrate for ectodomain shedding? To answer this question requires contemplation of the physiological function of APP. APP is a type 1 integral protein that is subjected to a host of post-translational processing events, including N- and O-glycosylation, tyrosyl sulfation, phosphorylation, and proteolysis [39,43,47,48]. Most (60%–80%) newly synthesized APP is subjected to terminal intracellular degradation that generates no discrete fragments [5]. A smaller fraction of APP molecules (approximately 20% in PC12 cells under basal conditions [5]) undergoes ectodomain shedding catalyzed by either the α-secretase (nonamyloidogenic) or β-secretase (potentially amyloiodgenic) pathway. When PKC is activated, the stoichiometry of shed sAPP_α_ rises from 2 mol shed per 10 mol synthesized to 4 mol shed per mole synthesized. Most of this shedding is catalyzed by the α-secretase pathway, but a trace amount (<5% [49]) is catalyzed by β-secretase/β-site APP cleaving enzyme [50]. sAPP_α_ and sAPP_β_ differ by the inclusion in sAPP_α_ of the first 16 residues of Aβ. Unlike sAPP_α_, which is generated at the plasma membrane, most sAPP_β_ is probably generated by cleavage within the *trans*-Golgi network and endocytic pathway vesicles. HoloAPP levels are likely limiting at one or more sorting steps in the late secretory pathway, since activated sAPP_α_ shedding is apparently accompanied by diminished generation of sAPP_β_ [51].

What is the function of shed sAPP_α_? Again, from other molecules, we know that shedding can serve important cellular functions by releasing diffusible ligands from their membrane-bound precursors (e.g., TGFα and TNFα) or by terminating intercellular signaling (e.g., Notch). A popular model holds that sAPP_α_ may function as a neurotrophic and/or neuroprotective factor, and may promote neurite outgrowth [52]. More recent evidence suggests that released APP derivatives modulate efficacy of neurotransmission at the synapse [53]. Targeted deletion of APP has not revealed a striking phenotype [54], presumably because of functional redundancy supplied by APP-like proteins [55]. Mice with double and triple null mutations in various combinations of APP, APP-like protein 1, and APP-like protein 2 are now being created, in search of evidence for a definitive function for APP.

Cao and Sudhof [56] have recently discovered that the APP C-terminal fragment generated by α- or β-secretase is itself cleaved to release Aβ and an APP intracellular domain (AICD) that diffuses into the nucleus, possibly acting there as a transcription factor. The pathway leading to AICD must be initiated by ectodomain shedding: holoAPP cannot directly give rise to AICD. Therefore, one important function for α- and/or β-secretase processing of APP may be the eventual generation of AICD. Our results suggest that Rho/ROCK signaling provides modulation of basal and stimulated α-secretase activity. It will now be important to dissect pathways upstream of Rho/ROCK signaling in order to identify the intracellular and intercellular events that participate in Rho/ROCK regulation of α-secretase under physiological and pathological conditions.

The potential role of cholesterol in α-secretase-mediated shedding was discovered by Bodovitz and Klein [57] who used β-cyclodextrin to lower cellular cholesterol. Kojro et al. [15] confirmed this observation, using not only β-cyclodextrin but also lovastatin to lower cellular cholesterol. These investigators proposed that elevated ADAM-10 activity and protein levels contributed to these effects. These basic observations dovetailed with emerging epidemiological evidence that administration of statins might lead to a diminished incidence of Alzheimer disease [8,9,10,11]. Despite this, however, the association of statins and cholesterol levels with activated α-secretase-mediated shedding of the APP ectodomain was unexpected and not readily explicable by existing knowledge regarding regulation of α-secretase activity. The best characterized regulation of α-secretase processing typically involves protein phosphorylation via PKC [5,29] or ERKs [7] or protein dephosphorylation by protein phosphatase 1 or 2A [29]. We recently excluded the possibility that either PKC or ERK plays a role in statin-activated shedding [16], raising the possibility that other protein phosphorylation signaling pathways might link statins and/or cholesterol to α-secretase activation.

Maillet et al. [58] implicated the Rho pathway in modulation of α-secretase activity while dissecting the activated shedding process that accompanies serotonergic signal transduction. These investigators discovered that Rap1 acts through Rac to modulate α-secretase processing of APP. Soon thereafter, ROCK1 was discovered by Zhou et al. [28] to modulate a downstream processing step in APP metabolism that involves presenilin/γ-secretase-mediated proteolysis of APP C-terminal fragments C99 and C83. These investigators discovered that activation of ROCK1 may account for how nonsteroidal anti-inflammatory drugs specify the scissile bond within the APP transmembrane domain that is cleaved by presenilin/γ-secretase to generate the C-terminus of Aβ. Based on these reports, we asked whether the Rho/ROCK pathway might play a role in controlling shedding of sAPP_α_ following statin application.

CA ROCK1 and DN ROCK1 molecules yielded direct and complementary evidence that ROCK1 was indeed a candidate for modulation of statin-activated α-secretase action. Further, we were able to demonstrate that α-secretase activity could be modulated by molecules further upstream in the isoprenoid pathway (see Figure 7). FTI-1, an inhibitor of farnesyl transferase also known as L-744,832 [59], mimicked and potentiated statin-activated shedding, presumably by blocking transfer of isoprenoid moieties to a Rho protein by farnesyl transferase, and thereby decreasing Rho activity. However, FTI-1 treatment can also increase the level of geranylgeranylated isoforms of certain Rho proteins, e.g., the inhibitory geranylgeranylated RhoB protein [60]. In further support of a role for isoprenoids, we were able to demonstrate that supplementation of cells with mevalonate abolished statin-activated shedding (see Figure 4). Statins block HMGCoA reductase generation of mevalonate from 3-hydroxy-methyl-glutarate (see Figure 7). Therefore, the addition of mevalonate would be predicted to antagonize statin action via the isoprenoid pathway, by relieving statin-induced mevalonate deficiency. As predicted by this model, we observed that statin-activated shedding was abolished by adding mevalonate. Taken together, these results suggest the existence of a reciprocal relationship between isoprenoid-mediated Rho/ROCK signaling and sAPP_α_ shedding, i.e., activation of ROCK1 blocks basal and stimulated shedding while ROCK1 inhibition apparently relieves a tonic negative influence exerted on shedding by ROCK1 activity.

As in PKC- and ERK-activated shedding, the ROCK1 substrate effector molecule or molecules that regulate proteolysis by ADAMs remain to be identified. The cytoplasmic domains of both APP and ADAM-17 have been evaluated as candidates for important targets of protein phosphorylation during the regulated shedding process, but neither “substrate activation” nor “enzyme activation” appears to explain the phenomenon, i.e., phosphorylation of neither APP nor ADAM-17 dramatically increases the efficiency of α-secretion [61,62], indicating that activation is more indirect.

Our data using statins and PO localize the mediator of statin-activated shedding to the plasma membrane, upstream of endocytosis, as appears to be the case for PKC-activated shedding [36,63,64,65]. Similar conclusions were drawn by Bosenberg and colleagues [36] who used streptolysin-porated cells and N-ethylmaleimide to demonstrate that reconstitution of activated shedding of TGFα from CHO cells does not require membrane trafficking and apparently occurs on the plasma membrane. These results suggest that a tightly membrane-associated regulatory subunit of the α-secretase complex is likely to be the key phosphoprotein that mediates α-secretase activity as a function of its state of phosphorylation by PKC and perhaps also ERK and ROCK1. The molecular identity of this phosphoprotein remains unknown.

α-Secretase activation is a potential therapeutic strategy for modifying cerebral amyloidosis in Alzheimer disease [66]. This proposal is supported by recent evidence that either genetic modification of ADAM-10/α-secretase activity [67] or administration of bryostatin, a PKC activator [68], can modulate levels of brain Aβ in plaque-forming transgenic mice. α-Secretase activation may explain how statins lower the risk for Alzheimer disease [69], since atorvastatin diminishes Aβ burden in plaque-forming transgenic mice [13]. If α-secretase stimulation is to be truly viable as a human clinical intervention, it will be essential to assess the possibility that enhanced APP ectodomain shedding might incur mechanism-based toxicity (analogous to the concerns currently surrounding γ-secretase inhibitors). Along this line, extension of this work to other shed proteins will be important to determine the impact of enhanced shedding via ADAM proteinases on other substrates of those proteinases, including Notch, pro-TGFα, pro-TNFα, and CD44 [3].

Preliminary results from a pilot proof-of-concept using atorvastatin in a human clinical treatment trial are consistent with the proposed beneficial effects of this class of compounds [70]. Since atorvastatin has low blood–brain barrier permeability [71], this beneficial effect, if attributable to Aβ lowering, must be due to altered Aβ metabolism in the periphery. Reduction in levels of free Aβ in the circulation has been demonstrated to lead to diminution in brain plaque burden following active or passive immunization [72]. It is conceivable that, if statins lower circulating Aβ, this effect could secondarily cause diffusion of central nervous system interstitial Aβ down its concentration gradient and into the cerebrospinal fluid and circulation, from which it is cleared. To date, however, this mechanism is not supported by data from human clinical trials, where statin administration has shown no consistent effect on levels of circulating or cerebrospinal fluid Aβ [73].

The results reported here point to several areas for additional investigation. As described above, the key substrate or substrates linking cytoplasmic protein phosphorylation to intralumenal or cell-surface protelysis remain to be identified. Nonetheless, α-secretase activation has been validated as a viable therapeutic strategy for modulating cerebral amyloidosis [67]. Identification of the role of the Rho/ROCK pathway in regulating α-secretase provides a new avenue for its therapeutic activation, even though the potential relevance of atorvastatin-mediated ROCK1 inhibition in neurons may not explain the apparent clinical benefits of the drug. Still, if the reported disease-modifying effect of atorvastatin is confirmed in the National Institute on Aging's large, multi-center trial of simvastatin, one or more compounds of this class may be among the first disease-modifying compounds approved by the Food and Drug Administration for slowing the progression of Alzheimer disease.

## Supporting Information

### Accession Numbers

The GenBank (http://www.ncbi.nlm.nih.gov/Genbank/) accession numbers for the proteins discussed in this paper are ROCK1 (NP_005397) and APP695, 595–596 KM/NL (NP_958817).

**Patient Summary**

**Background**

Large-scale studies have found a link between taking cholesterol-lowering drugs called statins and a decreased risk of developing Alzheimer disease. But it is not clear why statins might protect people from getting the disease. The brains of people who have died from Alzheimer disease show remnants of damaged cells called “tangles” as well as “amyloid plaques” in the spaces between the cells. These plaques are mostly made up of collections of a protein called amyloid-beta. It is the buildup of this protein that is thought to cause the brain damage and dementia associated with Alzheimer disease. The protein itself is formed when another, larger protein called APP (Alzheimer amyloid-beta peptide precursor protein) is broken down (or cleaved). There are two ways in which APP can be broken down. “Bad cleavage” releases the toxic amyloid-beta, whereas “good cleavage” destroys it. When researchers gave statins to animals over a long period of time, they found that statins could slow down the formation of amyloid plaques. From the animal experiments, it seemed that statins somehow caused more good cleavage to occur.

**Why Was This Study Done?**

This study examined how statins can affect APP cleavage.

**What Did the Researchers Do?**

They studied cells to see which of the players known to be involved in APP cleavage were affected by statin.

**What Did They Find?**

Statin's ability to promote “good cleavage” of APP involves a molecular pathway called the Rho/ROCK1 pathway. It seems that when ROCK1 is active, less good cleavage takes place. But in the presence of statins, ROCK1 is less active, shifting the balance toward good cleavage. Consistent with this, when the scientists blocked the Rho/ROCK1 pathway, they saw the good cleavage pattern even without statin.

**What Does This Mean for Patients?**

Inhibition of the Rho/ROCK1 pathway could explain some of the beneficial effects of statins against Alzheimer disease. And the pathway itself seems worth more research to see whether it might be a good target for new ways to prevent and treat Alzheimer disease.

**What Are the Limitations of the Study?**

Statins are likely to influence the risk for Alzheimer disease by several different pathways, and future studies will need to show how important this particular pathway is in the overall picture. Moreover, studies like this one are by necessity done in cells under carefully controlled laboratory conditions and still a long way from the development of safe and effective drugs.

**More Information Online**

Factsheet on statins from the Alzheimer's Association: http://www.alz.org/Resources/TopicIndex/statins.asp

General information at the Alzheimer's Disease Education and Referral Center at the United States National Institute of Aging: http://www.alzheimers.org/index.html

Homepage of Alzheimer's Disease International, an umbrella organization of Alzheimer disease associations around the world: http://www.alz.co.uk/

## Abbreviations

ADAM: a disintegrin and metalloproteinase
AICD: APP intracellular domain
APP: Alzheimer amyloid-β peptide precursor protein
Aβ: amyloid-β peptide
CA: constitutively active
csAPP: cell-surface Alzheimer amyloid-β peptide precursor protein
DN: dominant negative
ERK: extracellular-signal-regulated protein kinase
FTI-1: farnesyl transferase inhibitor
GFP: green fluorescent protein
PKC: protein kinase C
PO: phenylarsine oxide
ROCK: Rho-associated protein kinase
sAPP_α_: α-secretase-cleaved soluble Alzheimer amyloid-β peptide precursor protein ectodomain
TGFα: transforming growth factor α
TNFα: tumor necrosis factor α
